# Unveiling new insights: a comprehensive questionnaire-based single-center study of sebaceous nevus

**DOI:** 10.3389/fonc.2025.1529249

**Published:** 2025-09-25

**Authors:** Jiamin Jin, Tao Dai, Lu An, Bohan Lai, Jieyu Gu, Boxuan Wei, Shen Wang, Feng Xie

**Affiliations:** ^1^ Department of Plastic and Reconstructive Surgery, Shanghai Ninth People’s Hospital, Shanghai Jiao Tong University School of Medicine, Shanghai, China; ^2^ Department of Wound Reconstructive Surgery, Tongji Hospital of Tongji University, Shanghai, China; ^3^ Department of Burn and Plastic Surgery, Huai’an 82 Hospital, Huaian, Jiangsu, China

**Keywords:** sebaceous nevus, clinical features, familial factors, quality of life, treatment, COVID-19

## Abstract

**Introduction:**

Sebaceous nevus (SN) is a rare, benign, congenital tumor. There is limited information on its clinical features, familial influences, quality of life (QoL) impact, and treatment outcomes in Chinese patients. This study aimed to comprehensively examine these aspects.

**Methods:**

A questionnaire-based survey was conducted among patients with SN who visited the Department of Plastic Surgery at Shanghai Ninth People’s Hospital between January 2018 and December 2023. Healthy control children were recruited using the ‘best friends’ principle to assess familial influences. QoL impact was measured using the Children’s Dermatology Life Quality Index (CDLQI).

**Results:**

Our analysis revealed that the severity of skin-related symptoms correlated with the extent of hyperplasia and lesion size. Maternal factors during pregnancy—including medication use, secondhand smoke exposure, toxic chemical contact, and illness (notably COVID-19 infection)—were significantly associated with a higher incidence of SN. Most patients reported a mild to moderate QoL impact. Surgical treatment demonstrated higher satisfaction rates compared to laser therapy.

**Discussion:**

These findings suggest that hyperplasia and lesion size may predict symptom severity. The observed association between maternal COVID-19 infection and the occurrence of SN warrants further investigation. Surgical intervention appears to be an effective treatment, particularly for easily removable lesions.

## 1 Introduction

Sebaceous nevus (SN), a rare kind of birthmark, occurs in approximately 1 in 1000 individuals, predominantly found on the head and neck regions (1, 2). SN lesions on the scalp often result in alopecia (3). A subset of SN, termed “large SN”, covers more than 1% of the body area, posing challenges in removal (4).

In our outpatient clinic, while some SN patients complain about disturbing symptoms like pruritus and bleeding, others remain asymptomatic. There is a pressing need to investigate the predictive phenotypic factors for symptoms.

Previous research has suggested that maternal factors during pregnancy may influence the risk of congenital skin diseases (5). However, their potential impact on SN development remains unexplored.

SN is driven by somatic mutations in genes like HRAS and KRAS (6). Nevertheless, six familial SN cases have been documented (7–12), yet studies investigating familial SN in Asian cohorts are lacking.

It is well-established that various skin diseases, such as acne and atopic dermatitis, significantly impact patients’ quality of life (QoL) (13, 14). Given the cosmetic concerns, treatment challenges due to recurrent lesions, associated symptoms, and the QoL of SN patients warrant attention.

Optimal SN management remains unclear, with no consensus on surgical interventions (15). Laser therapy, though commonly used, carries a nonnegligible recurrence rate (16). Patient satisfaction with different treatments (surgery versus laser therapy) has not been adequately studied.

To address these gaps, we initiated a retrospective study investigating SN epidemiology, clinical features, familial factors, QoL impact, and treatment outcomes. This research aims to provide insights into the etiology and pathogenesis of SN.

## 2 Materials and methods

### 2.1 Questionnaire design

Our case questionnaire comprised four sections:

#### 2.1.1 Clinal characteristics

This section recorded age of onset, SN hyperplasia status, complications, and skin-related symptoms (numbness, dryness, pruritus, pain, burning sensation, bleeding, and swelling). Symptoms were rated on a 4-point scale (0 = none; 1 = mild; 2 = moderate; 3 = severe).

#### 2.1.2 Family factors

This part assessed maternal pregnancy factors and family history of patients. Additionally, for patients born after 2020, we included a question regarding any COVID-19 infection during their mother’s pregnancy.

#### 2.1.3 Quality of life assessment

We utilized the Children’s Dermatology Life Quality Index^©^ (CDLQI) to evaluate the impact of the skin disease on QoL of children patients (17). Ten items addressed QoL effects over the preceding week, scored on a 4-point Likert scale from ‘not at all’ (0) to ‘very much’ (3). Total scores were categorized: 0–1 (no effect), 2–6 (small effect), 7–12 (moderate effect), 13–18 (very large effect), and 19–30 (extremely large effect) (18).

#### 2.1.4 Treatment satisfaction

This section documented post-treatment recurrence and satisfaction scores (0 = Dissatisfied; 1 = Somewhat dissatisfied; 2 = Acceptable; 3 = Satisfied).

The control questionnaire exclusively addressed maternal pregnancy factors.

### 2.2 Patients group

We conducted a nationwide questionnaire-based epidemiological survey of SN patients presenting to the Department of Plastic Surgery, Shanghai Ninth People’s Hospital, between 1 January 2018 and 31 December 2023. Diagnoses were confirmed by two plastic surgeons. The cohort comprised 204 patients with isolated SN and 5 with concomitant SN and verrucous epidermal nevus (VEN). Written informed consent was obtained from all participants, their parents, or their legal guardians who completed the questionnaires. This study was approved by the IRB of Shanghai Ninth People’s Hospital (approval No. 201743). The details of 5 patients were shown independently. For patients with isolated SN, we recorded demographic data, lesion localization, size, and pathological diagnosis. Among this cohort, 74 participants provided only demographic data and basic SN characteristics (size, onset site, location). Of the remaining patients, 130 completed both Part 1 (clinical characteristics) and family history questionnaires, with 104 able to recall maternal pregnancy events. Additional questions regarding maternal COVID-19 infection were answered by families of 53 patients born after 2020. Furthermore, 43 children aged 4–18 years with partially treated or untreated SN completed quality of life assessments. For treatment outcome analysis, we selected 31 patients with comparable lesion size and location who underwent either surgical excision (n=16) or laser therapy (n=15) to evaluate treatment satisfaction.

### 2.3 Controls group

Using demographic data from the 104 patients reporting maternal events, we recruited controls via the ‘best friends’ principle (individuals without SN or SN family history). We collected 136 control questionnaires, including 72 from individuals born after 2020.

### 2.4 Statistical analysis

The questionnaire data were managed in Excel and analyzed using R software (version 4.3.0). Continuous variables are reported as mean ± SD; categorical variables as frequencies (percentages). Regional distribution mapping used the ‘hchinamap’ package (v0.1.0). Case-control comparisons employed ‘epiDisplay’ (v3.5.0.2), calculating odds ratios (ORs) with 95% confidence intervals (CIs) for categorical variables.

## 3 Results

### 3.1 Demographic data

**Table 1** presents the demographic and baseline clinical characteristics of 204 sebaceous nevus (SN) patients. The cohort showed female predominance (116 females vs. 88 males; ratio 1:1.32). Notably, secondary neoplasms developed in two patients: syringocystadenoma papilliferum in a 34-year-old male and sebaceoma in a 65-year-old female.

**Table 1:** Characteristics of 204 single sebaceous nevus patients.

Characteristics	Number (%)
Gender	
Male	88(43.1%)
Female	116(56.9%)
Onset site	
Face	89 (43.6%)
Frontal Region	16 (7.8%)
Orbital Region	14 (6.9%)
Labial Region	5 (2.5%)
Nasal Region	14 (6.9%)
Infraorbital Region	10 (4.9%)
Buccal Region	8 (3.9%)
Mandibular Region	10 (4.9%)
Multiregions	12 (5.9%)
Scalp	71 (34.8%)
Vertex	15 (7.4%)
Lateral Cranium	39 (19.1%)
Occiput	17 (8.3%)
Neck	17 (8.3%)
Face + Scalp	6 (2.9%)
Face + Neck	7 (3.4%)
Face + Scalp + Neck	1 (0.5%)
Limbs	1 (0.5%)
Trunk	1 (0.5%)
Unknown	11 (5.4%)
Size (percentage of body area)	
<1%	167 (81.9%)
>=1%	26 (12.7%)
Unknown	11 (5.4%)
Lesion locations	
Unilateral	186 (91.2%)
Bilateral	7 (3.4%)
Unknown	11 (5.4%)
Age at first visit	
<10 years	116 (56.8%)
10–18 years	42 (20.6%)
>18 years	44 (21.6%)
Unknown	2 (1.0%)
The pathological report	
None	102 (50%)
Yes	
Normal	99 (49.0%)
With second neoplasm	
Benign	2 (1.0%)
Malignant	0 (0.0%)
Total	204 (100)

Unknown: Reported data omitted specific details.

### 3.2 Clinical features

Detailed clinical data for 130 patients are presented in **Supplementary Table S1**, with most residing in Chinese coastal regions (**Supplementary Figure S1**). **Supplementary Table S2** outlines the onset time and hyperplasia of lesions after birth in these patients, specifically. Notably, 3.1% (4/130) exhibited bilateral facial lesions, and 11.5% (15/130) developed SN after age 1 year. Hyperplasia occurred in 17.7% (23/130) of patients, manifesting as noticeable color deepening, accelerated growth relative to the body, and protrusion above the skin surface. And patient-reported triggers included puberty, mechanical irritation (scratching/rubbing), spicy foods, seasonal changes, and sun exposure.

**Supplementary Table S3** delineates the frequencies of skin-related symptoms attributed to SN, with the top three incidences observed for itchiness, dryness, and bleeding. Statistical analysis revealed males demonstrated significantly higher pruritus risk compared to females (OR 0.46, 95% CI 0.22–0.93; p=0.03). The total skin symptom scores correlated positively with lesion size (p=0.029) and hyperplasia presence (p=0.033), but not with age, location, or onset time. Larger lesions increased risks of dryness (p=0.024), numbness (p=0.042), and bleeding (p=0.047). Hyperplasia showed significant associations with older age (OR: 5.18, 95%CI: 1.65~16.22, p<0.001), swelling symptoms (OR: 3.42, 95%CI: 1.1~10.66, p=0.038), larger lesion size (OR: 4.76, 95%CI: 1.55~14.6, p=0.009), and bilateral involvement (p<0.001). Refer to **Supplementary Tables S4**–**S7** for detailed data.

Concurrent birthmarks occurred in 6.2% (8/130) of patients, including hyperpigmentation, congenital melanocytic nevi, and hemangiomas. Allergic disease history was present in 9.2% (12/130), consistent with general population rates (19–21). No systemic abnormalities were documented.

### 3.3 Family factors

This section encompasses maternal pregnancy factors and familial history.

#### 3.3.1 Maternal pregnant factors

We compared maternal pregnancy exposures between 104 SN patients and 136 controls (**Supplementary Table S8**). Mothers of SN patients reported significantly higher rates of medical complications during pregnancy compared to controls (55.8% vs. 41.9%; p=0.033), primarily threatened miscarriage, cold and hyperemesis gravidarum. Notably, among 125 children born after 2020 (53 cases, 72 controls), maternal COVID-19 infection was more frequent in the SN cohort (OR 4.78, 95% CI 1.82–12.53; p<0.001). Furthermore, the infection rate was found to be independent of other pregnancy-related diseases but correlated with a history of drug use and chemical exposure during pregnancy. Even after adjusting for these factors, the infection rate remained statistically significant (refer to **Supplementary Tables S9**, **S10**); however, this finding need validation with a larger cohort.

Medication use during pregnancy was significantly more frequent among mothers of SN patients compared to controls (28.8% vs. 9.6%; OR 3.84, 95% CI 1.88-7.82; p<0.001), with diverse agents employed for pregnancy-related conditions. The heterogeneity of medications precluded drug-specific analysis. Secondhand smoke exposure affected 23.1% of SN mothers versus 11.0% of controls (OR 2.43, 95% CI 1.20-4.92; p=0.02). Chemical exposures during pregnancy were reported by 11 SN mothers (primarily formaldehyde: 9/11) compared to one control mother (non-formaldehyde exposure; OR 15.97, 95% CI 2.03–125.8; p<0.001). In one notable case, a third-trimester ultrasound detected fetal facial spots, with corresponding bilateral facial SN lesions manifesting postnatally.

#### 3.3.2 Sebaceous nevus family history positive family

Three families demonstrated familial occurrence of SN or verrucous epidermal nevus (VEN). The family trees of these individuals are provided in the **Supplementary Figure S2**. **Supplementary Figure S3** illustrates epidermal nevus lesions observed in one of the families.

### 3.5 Quality of life

CDLQI assessment revealed variable quality of life impacts among SN patients (**Table 2**). Among the patients, 9.3% (4/43) reported a small effect (score 2-6), while an equal proportion reported a moderate effect (score 7-12). Significantly higher total scores correlated with male sex, lesions involving >1% body surface area (BSA), and head involvement. Specifically, males expressed more complaints than females in regards to ‘Q3’, ‘Q5’, and ‘Q6’. Patients with larger lesions were more susceptible to feelings of ridicule (‘Q8’) (p=0.005). Moreover, individuals with head involvement experienced greater impacts across all aspects compared to those with lesions on the neck. Refer to **Supplementary Tables S11**, **S12** for detailed data.

**Table 2:** Impact of sebaceous nevus on quality of life.

CDLQI questions	SN (N = 43)				
	Not at all (0)	Only a little (1)	Quite a lot (2)	Very much (3)	M (SD)
Q1. Itchy, ‘scratchy’, sore or painful skin	39/43 (90.7%)	3/43 (7.0%)	1/43 (2.3%)	0	0.12 (0.39)
Q2. Embarrassed, self-conscious, upset or sad because of skin	35/43 (81.4%)	6/43 (14.0%)	2/43 (4.7%)	0	0.23 (0.53)
Q3. Friendships	36/43 (83.7%)	5/43 (11.6%)	2/43 (2.3%)	0	0.21 (0.51)
Q4. Clothing decisions	38/43 (88.4%)	3/43 (7.0%)	2/43 (4.7%)	0	0.16 (0.48)
Q5. Going out, playing, or hobbies	37/43 (86.0%)	5/43 (11.6%)	1/43 (2.3%)	0	0.16 (0.43)
Q6. Swimming or other sports	39/43 (90.7%)	4/43 (9.3%)	0	0	0.09 (0.29)
Q7. Schoolwork/Holiday in past week	40/43 (93.0%)	3/43 (7.0%)	0	0	0.07 (0.26)
Q8. Calling names, teasing, bullying, asking questions, or avoiding	38/43 (88.4%)	4/43 (9.3%)	1/43 (2.3%)	0	0.14 (0.41)
Q9. Sleep	41/43 (95.3%)	1/43 (2.3%)	1/43 (2.3%)	0	0.07 (0.33)
Q10. Effect of treatment on quality of life	35/43 (81.4%)	8/43 (18.6%)	0	0	0.19 (0.39)
CDLQI total sum score					1.44 (2.87)

M, Mean; SD, Standard Deviation.

### 3.6 Treatment outcome

Out of the 31 patients, 7 underwent surgical treatment while 24 received laser therapy for their SN. The follow-up period ranged from 5 to 42 months, with a mean duration of 16 months. SN lesions in these patients comprised less than 1% of the body area and were deemed suitable for surgical removal. Analysis of satisfaction scores and recurrence rates is presented in **Table 3**. Notably, patients who underwent surgical resection reported higher satisfaction levels compared to those who received laser treatment.

**Table 3:** Comparison of satisfaction scores and recurrence rate between surgery and laser therapy.

Characteristics	Surgery	Laser	P value
Satisfaction scores			0.001 ***
Mean (SD)	2.43 (0.49)	1.54 (0.58)	
Recurrence			0.009 **
No	7 (100)	10 (41.7%)	
Yes	0 (0)	14 (58.3%)	
Total	7 (100%)	24 (100%)	

The number of asterisks (*) denotes the level of statistical significance: * for p < 0.05, ** for p < 0.01, and *** for p < 0.001.

### 3.7 Five patients with concomitant SN and VEN

The details of the five patients are presented in **Table 4** and **Supplementary Figure S4**. Among them, Case 4 showed abnormal ocular pigmentation and scoliosis. Multi-point biopsies were conducted for the lesion of Case5, with results illustrated in **Supplementary Figure S5**. The central region exhibited characteristics consistent with SN, while the peripheral area displayed features indicative of VEN.

**Table 4:** Details of 5 patients with concomitant SN and verrucous epidermal nevus.

Characteristics	Case1	Case2	Case3	Case4	Case5
Gender	Female	Male	Female	Female	Female
Age at first visit	26 y	3 y	13 y	15 y	17 y
Age of Onset	At birth	<1 years	<1 years	At birth	< 1 years
Site	Face& Trunk& limbs	Face& Trunk	Trunk& limbs	Face& Trunk	Face
Locations	Bilateral	Unilateral	Unilateral	Bilateral	Unilateral
Family histroy	None	None	None	None	None
Complication	None	None	None	**Scoliosis**	None

Scoliosis: a medical condition characterized by an abnormal lateral (side-to-side) curvature of the spine, often forming an “S” or “C” shape.

## 4 Discussion

This comprehensive investigation delineates epidemiological patterns, clinical manifestations, familial factors, quality of life impacts, and treatment outcomes in sebaceous nevus (SN) patients. We observed significant female predominance (male:female = 1:1.32), contrasting with near-equal gender distributions in prior reports (22–24) but similar to the verrucous epidermal nevus (VEN) epidemiology research (25).

Patient-report hyperplasia triggers included puberty, mechanical friction, UV exposure, seasonal variation, and spicy foods. Statistical analyses confirmed symptom severity correlates with lesion size and hyperplasia presence, while hyperplasia itself associates with advanced age, larger lesions, and bilateral distribution—unstable lesions demonstrating heightened swelling symptoms. Although pubertal androgen surges may drive SN proliferation (26) and UV exposure has been implicated in secondary tumors (27), we found no direct hyperplasia-neoplasm association.

Regarding scratching friction, climate, and diet, further investigation is warranted to determine their impact on SN progression. S Pradhan demonstrated the existence of *Malassezia globosa* on a 3-month-old SN case infiltrated heavily by immune cells (28). This suggests a potential role for microbes in causing itching and promoting SN hyperplasia. Based on these findings, clinical practice may involve instructing patients to refrain from scratching SN lesions and consuming spicy foods, initiating interventions before puberty (29), and implementing sun protection measures.

Compared to controls, mothers of SN patients exhibited a higher incidence of pregnancy-related diseases, second-hand smoking exposure, medication use, and exposure to chemical substances (notably formaldehyde). Prenatal ultrasonography was found to have some efficacy in detecting large SN, although its role in early detection remains limited (30, 31). Interestingly, while there was no statistical difference in the incidence of common influenza, mothers of SN infants born during the COVID-19 pandemic showed a higher likelihood of COVID-19 infection during pregnancy compared to controls (OR 4.78, p<0.001). Whether this observation is incidental requires validation in a larger cohort. HPV has been detected in SN samples, implicating its potential role in SN development through the integration of HPV DNA into the host (32–34). HIV-infected patients have also been noted to develop SN-like lesions (35). However, the effect of COVID-19 on SN remains unknown. One hypothesis suggests that placental infection may lead to dysplasia (36) and fetal distress, thereby promoting somatic mutations (37). Another possibility is that intrauterine COVID-19 infection disrupts skin development, although no SN lesions have been reported post-COVID-19 infection (38). Unfortunately, patients whose mothers were infected with COVID-19 during pregnancy were not tested COVID-19 at birth, complicating making it challenging to determine whether the adverse uterine environment caused by COVID-19 infection or fetal infection itself impacts SN occurrence. While studies on the impact of COVID-19 infection on fetuses typically focus on severe adverse outcomes such as death and deformities (39, 40), its association with the incidence of body surface tumors has not been extensively explored. Therefore, our study represents the first to report a potential link between maternal COVID-19 and the development of fetal body surface tumors.

There were 3 cases with a positive family history of SN or VEN, whose inheritance did not conform to Mendelian inheritance patterns. Previous studies have demonstrated that SN is driven by somatic mutations in genes such as HRAS and FGFR3. However, homozygous mutations of those genes are typically lethal (41) and cannot be inherited by offspring or exhibit familial aggregation. Conducting genome-wide association studies (GWAS) (42) on large cohorts of the SN population may help identify SNP sites associated with SN and provide a foundation for prenatal screening and targeted drug treatments for large SN lesions.

For patients with SN presenting similar lesions, those undergoing surgical treatment reported higher satisfaction compared to those undergoing laser treatment. Given the progressive nature of SN in adolescents, we advocate early surgical removal for amenable lesions before puberty. Among patients treated with laser therapy, the recurrence rate was 14/24, which was lower than the 5/6 recurrence rate reported in previous study (16), though three patients experienced larger lesions post-treatment recurrence.

Regarding patients with both SN and VEN, we support the hypothesis suggesting that they are variants of each other (19 While Case 5 biopsies demonstrated distinct zonal histology (central SN vs. peripheral VEN), we observed no transitional morphology as described by Hafner (43). This phenomenon may be attributed to the stimulation of SN by the dilator, with epidermal hyperplasia preceding sebaceous hyperplasia. It has been suggested that mutant cells at the edge reach the end of differentiation and are unable to differentiate into sebaceous glands (44). Moving forward, dual-phenotype lesions will undergo DNA sequencing to confirm clonal origins and homology.

A limitation of this study is that, the gender dimensions of patients was simply divided into male and female based solely on the visible external anatomy.

## Data Availability

The raw data supporting the conclusions of this article will be made available by the authors, without undue reservation.
