# Supplementary material for: Unveiling new insights: a comprehensive questionnaire-based single-center study of sebaceous nevus
Source: Front Oncol. 2025 Sep 25;15:1529249. doi: 10.3389/fonc.2025.1529249 (PMC12507612; doi:10.3389/fonc.2025.1529249)
Supplement: Supplementary file 1 [file DataSheet1.docx]

**Supplementary table 1.** Characteristics of 130 single sebaceous nevus patients who completed the first part of questionnaire.

|  | **Number (%)** |
| --- | --- |
| **Age** |  |
| Mean (SD) | 9.27 (11.67) |
| **Onset site** |  |
| Face | 60 (46.2%) |
| Scalp | 53 (40.8%) |
| Neck | 10 (7.7%) |
| Face + Scalp | 3 (2.3%) |
| Face + Neck | 1 (0.8%) |
| Face + Scalp + Neck | 1 (0.8%) |
| Limbs | 1 (0.8%) |
| Trunk | 1 (0.8%) |
| **Size (percentage of body area)** |  |
| <1% | 114 (87.7%) |
| >=1% | 16 (12.3%) |
| **Locations** |  |
| Unilateral | 126 (96.9%) |
| Bilateral | 4 (3.1%) |
| **Age at first visit** |  |
| 0-10 years | 84 (64.6%) |
| 10-18 years | 25 (19.2%) |
| >18 years | 21 (16.2%) |

**Supplementary table 2.** Detailed Clinical features of 130 single sebaceous nevus.

|  | **Number (%)** |
| --- | --- |
| **Age of Onset** |  |
| At birth | 99 (76.2%) |
| <1 years | 16 (12.3%) |
| 1~10 years | 7 (5.4%) |
| At puberty | 8 (6.1%) |
| **Hyperplasia** |  |
| No | 107 (82.3%) |
| Yes | 23 (17.7%) |
| **Epidermal nevus history** |  |
| No | 127 (97.7%) |
| Yes | 3 (2.3%) |

**Supplementary table 3.** Frequency of skin-related symptoms

| **Variable** | **Sebaceous nevus (N = 130)** | | | | |
| --- | --- | --- | --- | --- | --- |
|  | **No problem (0)** | **Moderate (1)** | **Severe (2)** | **Very severe (3)** | **M (SD)** |
| **Numbness** | 118/130 (90.8%) | 8/130 (6.2%) | 2/130 (1.5%) | 2/130 (1.5%) | 0.14 (0.49) |
| **Dryness** | 103/130 (79.2%) | 17/130 (13.1%) | 7/130 (5.4%) | 3/130 (2.3%) | 0.31 (0.68) |
| **Itchiness** | 77/130 (59.2%) | 34/130 (26.2%) | 14/130 (10.8%) | 5/130 (3.8%) | 0.59 (0.83) |
| **Pain** | 116/130 (89.2%) | 11/130 (8.5%) | 2/130 (1.5%) | 1/130 (0.8%) | 0.14 (0.44) |
| **Burning** | 120/130 (92.3%) | 7/130 (5.4%) | 2/130 (1.5%) | 1/130 (0.8%) | 0.11 (0.42) |
| **Bleeding** | 112/130 (86.2%) | 13/130 (10.0%) | 2/130 (1.5%) | 3/130 (2.3%) | 0.2 (0.57) |
| **Swelling** | 114/130 (87.7%) | 12/130 (9.2%) | 2/130 (1.5%) | 2/130 (1.5%) | 0.17 (0.51) |
| **Skin related symptom total sum score** |  |  |  |  | 1.65 (3.02) |

**Supplementary table 4.** The correlations between predictor variables and skin-related symptoms’ score

| **System score** | **Mean (SD)** | **p value** |
| --- | --- | --- |
| **Gender** |  | 0.2258 |
| Male | 2.03 (2.99) |  |
| Female | 1.38 (3.05) |  |
| **Age at first visit** |  | 0.7173 |
| Child | 1.49 (2.67) |  |
| Teenage | 2.24 (4.57) |  |
| Adult | 1.62 (2.04) |  |
| **Size** |  | 0.02944 * |
| <1% | 1.35 (2.30) |  |
| >=1% | 3.81 (5.78) |  |
| **Site** |  | 0.228 |
| Face | 1.12 (1.98) |  |
| Scalp | 2.13 (3.17) |  |
| Neck | 0.8 (1.32) |  |
| **Age of Onset** |  | 0.375 |
| At birth | 1.73 (3.06) |  |
| <1years | 1.44 (3.31) |  |
| 1-10years | 2.43 (3.74) |  |
| At purberty | 0.5 (0.926) |  |
| **Location** |  | 0.369 |
| Unilateral | 1.61 (2.98) |  |
| Bilateral | 3 (4.76) |  |
| **Hyperplasia** |  | 0.03347 * |
| No | 1.39 (2.81) |  |
| Yes | 2.87 (3.73) |  |

**Supplementary table 5.** The correlations between each skin-related symptom and size

| **Size** | **<1% (n=114)** | **>=1% (n=16)** | **OR (95%CI)** | **p value** |
| --- | --- | --- | --- | --- |
| **Numbness** |  |  | 4.42 (1.16~16.88) | 0.042 * |
| No | 106 (93) | 12 (75) |  |  |
| Yes | 8 (7) | 4 (25) |  |  |
| **Dry** |  |  | 3.66 (1.22~10.97) | 0.024 * |
| No | 94 (82.5) | 9 (56.2) |  |  |
| Yes | 20 (17.5) | 7 (43.8) |  |  |
| **Itch** |  |  | 1.53 (0.54~4.38) | 0.422 |
| No | 69 (60.5) | 8 (50) |  |  |
| Yes | 45 (39.5) | 8 (50) |  |  |
| **Pain** |  |  | 3.47 (0.94~12.78) | 0.072 ** |
| No | 104 (91.2) | 12 (75) |  |  |
| Yes | 10 (8.8) | 4 (25) |  |  |
| **Burn** |  |  | 3.53 (0.81~15.34) | 0.107 |
| No | 107 (93.9) | 13 (81.2) |  |  |
| Yes | 7 (6.1) | 3 (18.8) |  |  |
| **Bleed** |  |  | 3.53 (1.06~11.78) | 0.047 * |
| No | 101 (88.6) | 11 (68.8) |  |  |
| Yes | 13 (11.4) | 5 (31.2) |  |  |
| **Swell** |  |  | 2.83 (0.79~10.19) | 0.111 |
| No | 102 (89.5) | 12 (75) |  |  |
| Yes | 12 (10.5) | 4 (25) |  |  |

**Supplementary table 6.** The correlations between predictor variables and hyperplasia

| **Hyperplasia** | **No (n=107)** | **Yes (n=23)** | **OR (95%CI)** | **p value** |
| --- | --- | --- | --- | --- |
| **Age at first visit** |  |  | 5.18 (1.65~16.22) | <0.001 *** |
| Child | 77 (72) | 7 (30.4) |  |  |
| Teenage | 17 (15.9) | 8 (34.8) |  |  |
| Adult | 13 (12.1) | 8 (34.8) |  |  |
| **Size** |  |  | 4.76 (1.55~14.6) | 0.009 ** |
| <1% | 98 (91.6) | 16 (69.6) |  |  |
| >=1% | 9 (8.4) | 7 (30.4) |  |  |
| **Site** |  |  | 2.43 (0.53~11.18) | 0.47 |
| Face | 51 (49.5) | 9 (45) |  |  |
| Neck | 7 (6.8) | 3 (15) |  |  |
| Scalp | 45 (43.7) | 8 (40) |  |  |
| **Age of Onset** |  |  | 0.87 (0.18~4.24) | 0.019 * |
| At birth | 85 (79.4) | 14 (60.9) |  |  |
| <1years | 14 (13.1) | 2 (8.7) |  |  |
| 1-10years | 4 (3.7) | 3 (13) |  |  |
| At purberty | 4 (3.7) | 4 (17.4) |  |  |
| **Location** |  |  | - | <0.001 *** |
| Unilateral | 107 (100) | 19 (82.6) |  |  |
| Bilateral | 0 (0) | 4 (17.4) |  |  |
| **Numbness** |  |  | 1.63 (0.41~6.57) | 0.444 |
| No | 98 (91.6) | 20 (87) |  |  |
| Yes | 9 (8.4) | 3 (13) |  |  |
| **Dry** |  |  | 1.9 (0.69~5.24) | 0.257 |
| No | 87 (81.3) | 16 (69.6) |  |  |
| Yes | 20 (18.7) | 7 (30.4) |  |  |
| **Itch** |  |  | 1.76 (0.71~4.35) | 0.22 |
| No | 66 (61.7) | 11 (47.8) |  |  |
| Yes | 41 (38.3) | 12 (52.2) |  |  |
| **Pain** |  |  | 3.02 (0.91~10.07) | 0.074 |
| No | 98 (91.6) | 18 (78.3) |  |  |
| Yes | 9 (8.4) | 5 (21.7) |  |  |
| **Burn** |  |  | 3.54 (0.91~13.76) | 0.076 |
| No | 101 (94.4) | 19 (82.6) |  |  |
| Yes | 6 (5.6) | 4 (17.4) |  |  |
| **Bleed** |  |  | 2.79 (0.92~8.46) | 0.09 |
| No | 95 (88.8) | 17 (73.9) |  |  |
| Yes | 12 (11.2) | 6 (26.1) |  |  |
| **Swell** |  |  | 3.42 (1.1~10.66) | 0.038 * |
| No | 97 (90.7) | 17 (73.9) |  |  |
| Yes | 10 (9.3) | 6 (26.1) |  |  |

**Supplementary table 7.** The correlations between each variable and gender

| **Gender** | **Female (n=76)** | **Male (n=54)** | **OR (95% CI)** | **p value** |
| --- | --- | --- | --- | --- |
| **Age at first visit** |  |  | - | 0.865 |
| Mean | 9.12 | 9.48 |  |  |
| **Size** |  |  | 0.9 (0.31~2.59) | 0.848 |
| <1% | 67 (88.2) | 47 (87) |  |  |
| >=1% | 9 (11.8) | 7 (13) |  |  |
| **Site** |  |  | - | 0.913 |
| Face | 33 (47.1) | 27 (50.9) |  |  |
| Neck | 6 (8.6) | 4 (7.5) |  |  |
| Scalp | 31 (44.3) | 22 (41.5) |  |  |
| **Age of Onset** |  |  | - | 0.429 |
| At birth | 55 (72.4) | 44 (81.5) |  |  |
| <1years | 12 (15.8) | 4 (7.4) |  |  |
| 1-10years | 5 (6.6) | 2 (3.7) |  |  |
| At purberty | 4 (5.3) | 4 (7.4) |  |  |
| **Location** |  |  | 0.23 (0.02~2.24) | 0.307 |
| Unilateral | 75 (98.7) | 51 (94.4) |  |  |
| Bilateral | 1 (1.3) | 3 (5.6) |  |  |
| **Hyperplasia** |  |  | 0.59 (0.24~1.46) | 0.254 |
| No | 65 (85.5) | 42 (77.8) |  |  |
| Yes | 11 (14.5) | 12 (22.2) |  |  |
| **Numbness** |  |  | 0.69 (0.21~2.25) | 0.553 |
| No | 70 (92.1) | 48 (88.9) |  |  |
| Yes | 6 (7.9) | 6 (11.1) |  |  |
| **Dry** |  |  | 0.71 (0.3~1.67) | 0.434 |
| No | 62 (81.6) | 41 (75.9) |  |  |
| Yes | 14 (18.4) | 13 (24.1) |  |  |
| **Itch** |  |  | 0.46 (0.22~0.93) | 0.03 * |
| No | 51 (67.1) | 26 (48.1) |  |  |
| Yes | 25 (32.9) | 28 (51.9) |  |  |
| **Pain** |  |  | 0.35 (0.11~1.12) | 0.067 |
| No | 71 (93.4) | 45 (83.3) |  |  |
| Yes | 5 (6.6) | 9 (16.7) |  |  |
| **Burn** |  |  | 0.69 (0.19~2.51) | 0.74 |
| No | 71 (93.4) | 49 (90.7) |  |  |
| Yes | 5 (6.6) | 5 (9.3) |  |  |
| **Bleed** |  |  | 1.5 (0.53~4.28) | 0.449 |
| No | 64 (84.2) | 48 (88.9) |  |  |
| Yes | 12 (15.8) | 6 (11.1) |  |  |
| **Swell** |  |  | 0.68 (0.24~1.93) | 0.463 |
| No | 68 (89.5) | 46 (85.2) |  |  |
| Yes | 8 (10.5) | 8 (14.8) |  |  |

**Supplementary table 8.** Comparison of maternal pregnant factors (104 sebaceous nevus patients vs. 136 controls)

|  | **NS (n =104)** | **Control (n = 136)** | **OR (95% CI)** | **p value** |
| --- | --- | --- | --- | --- |
| **Age at first visit** |  |  | - | 0.783 |
| Mean (SD) | 4.91 (4.92) | 4.75 (4.05) |  |  |
| **Gender** |  |  | - | 0.11 |
| Female | 59 (56.7) | 63 (46.3) |  |  |
| Male | 45 (43.3) | 73 (53.7) |  |  |
| **Mother’s pregnant age** |  |  |  |  |
| **Mean (SD)** | 28.9 (4.06) | 28.5 (4.43) | - | 0.458 |
| **Grade** |  |  | 1.34 (0.59~3.07） | 0.483 |
| >=35 years | 10 (9.6) | 17 (12.5) |  |  |
| <35 years | 94 (90.4) | 119 (87.5) |  |  |
| **Pregnant disease** |  |  |  |  |
| **Two grade** |  |  | 1.75 (1.04~2.93) | 0.033 * |
| No | 46 (44.2) | 79 (58.1) |  |  |
| Yes | 58 (55.8) | 57 (41.9) |  |  |
| **Gestational diabetes** |  |  | 1.15 (0.4~3.29) | 0.788 |
| No | 97 (93.3) | 128 (94.1) |  |  |
| Yes | 7 (6.7) | 8 (5.9) |  |  |
| **Threatened abortion** |  |  | 2.83 (1.02~7.8) | 0.038 * |
| No | 92 (88.5) | 130 (95.6) |  |  |
| Yes | 12 (11.5) | 6 (4.4) |  |  |
| **Thyroid dysfunction** |  |  | 1.05 (0.4~2.76) | 0.921 |
| No | 96 (92.3) | 126 (92.6) |  |  |
| Yes | 8 (7.7) | 10 (7.4) |  |  |
| **Gestational hypertension** |  |  | 0.87 (0.14~5.3) | 1 |
| No | 102 (98.1) | 133 (97.8) |  |  |
| Yes | 2 (1.9) | 3 (2.2) |  |  |
| **Cold or flu** |  |  | 2.2 (0.91~5.29) | 0.074 |
| No | 90 (86.5) | 127 (93.4) |  |  |
| Yes | 14 (13.5) | 9 (6.6) |  |  |
| **Anemia** |  |  | 0.54 (0.21~1.37) | 0.189 |
| No | 97 (93.3) | 120 (88.2) |  |  |
| Yes | 7 (6.7) | 16 (11.8) |  |  |
| **Acute vomiting** |  |  | 2.02 (0.83~4.92) | 0.118 |
| No | 91 (87.5) | 127 (93.5) |  |  |
| Yes | 13 (12.5) | 9 (6.6) |  |  |
| **Drug consumption** |  |  | 3.84 (1.88~7.82) | < 0.001 *** |
| No | 74 (71.2) | 123 (90.4) |  |  |
| Yes | 30 (28.8) | 13 (9.6) |  |  |
| **Alcohol consumption** |  |  | - | 0.187 |
| No | 102 (98.1) | 135 (99.3) |  |  |
| Once | 2 (1.9) | 0 (0) |  |  |
| Always | 0 (0) | 1 (0.7) |  |  |
| **Smoking exposure** |  |  | 2.43 (1.2~4.92) | 0.02 * |
| No | 79 (76) | 120 (88.3) |  |  |
| Second-hand | 24 (23.1) | 15 (11) |  |  |
| Self | 1 (1) | 1 (0.7) |  |  |
| **Toxic chemical material exposure** |  |  | 15.97 (2.03~125.8) | < 0.001 *** |
| No | 93 (89.4) | 135 (99.3) |  |  |
| Yes | 11 (10.6) | 1 (0.7) |  |  |
| **Born after 2020** | NS (n=53) | Control (n=72) |  |  |
| **SARS-CoV-2 infection** |  |  | 4.78 (1.82~12.53) | < 0.001 *** |
| No | 35 (66) | 65 (90.3) |  |  |
| Yes | 18 (34) | 7 (9.7) |  |  |

**Supplementary table 9.** The correlations between SARS-CoV-2 infection and other pregnant factors

| **SARS-CoV-2 infection** | **No (n=100)** | **Yes (n=25)** | **OR (95%CI)** | **p value** |
| --- | --- | --- | --- | --- |
| **Gestational diabetes** |  |  | - | 0.599 |
| No | 94 (94) | 25 (100) |  |  |
| Yes | 6 (6) | 0 (100) |  |  |
| **Threatened abortion** |  |  | 0.78 (0.16~3.82) | 1 |
| No | 90 (90) | 23 (92) |  |  |
| Yes | 10 (10) | 2 (8) |  |  |
| **Thyroid dysfunction** |  |  | 1.54 (0.45~5.32) | 0.498 |
| No | 89 (89) | 21 (84) |  |  |
| Yes | 11 (11) | 4 (16) |  |  |
| **Gestational hypertension** |  |  | - | 1 |
| No | 98 (98) | 25 (100) |  |  |
| Yes | 2 (2) | 0 (0) |  |  |
| **Cold or flu** |  |  | 1.57 (0.38~6.4) | 0.459 |
| No | 92 (92) | 22 (88) |  |  |
| Yes | 8 (8) | 3 (12) |  |  |
| **Anemia** |  |  | 1 (0.26~3.85) | 1 |
| No | 88 (88) | 22 (88) |  |  |
| Yes | 12 (12) | 3 (12) |  |  |
| **Acute vomiting** |  |  | 2.84 (0.92~8.77) | 0.089 |
| No | 90 (90) | 19 (76) |  |  |
| Yes | 10 (10) | 6 (24) |  |  |
| **Drug consumption** |  |  | 3.84 (1.54~9.6) | 0.003 ** |
| No | 78 (78) | 12 (48) |  |  |
| Yes | 22 (22) | 13 (52) |  |  |
| **Alcohol consumption** |  |  | - | 0.2 |
| No | 100 (100) | 24 (96) |  |  |
| Once | 0 (0) | 1 (4) |  |  |
| **Smoking exposure** |  |  | - | 0.378 |
| No | 82 (82) | 18 (72) |  |  |
| Family | 16 (16) | 7 (28) |  |  |
| Self | 2 (2) | 0 (0) |  |  |
| **Toxic chemical material exposure** |  |  | 9.33 (1.6~54.34) | 0.016 * |
| No | 96 (96) | 20 (80) |  |  |
| Yes | 4 (4) | 5 (20) |  |  |

**Supplementary table 10.** The difference between SN mothers and control mothers of SARS-CoV-2 infection after adjusted by Drug consumption and Chemical material exposure

| **SARS-CoV-2 infection Stratified analysis by X** | **OR (95%CI)** | **p value** |
| --- | --- | --- |
| **X= Toxic drug consumption** |  |  |
| No | 5.31 (1.266~26.70) | 0.016 |
| Yes | 1.87 (0.333~13.72) | 0.478 |
| M-H combined | 3.45 (1.272~9.34) | **0.010** |
| **X= Chemical material exposure** |  |  |
| No | 3.67 (1.2199~12.0) | 0.01145 |
| Yes | - | 0.44444 |
| M-H combined | 4.00 (1.4733~10.9) | **0.00473** |

**Supplementary table 11.** The correlations between predictor variables and QoL

| **Qol score** | **Mean (SD)** | **p value** |
| --- | --- | --- |
| **Gender** |  | 0.1781 |
| Male | 2.18 (3.24) |  |
| Female | 0.962 (2.55) |  |
| **Age at first visit** |  | 0.8575 |
| Child | 1.52 (3.16) |  |
| Teenage | 1.36 (2.65) |  |
| **Size** |  | 0.111 |
| <1% | 1.03 (2.30) |  |
| >=1% | 3.57 (4.54) |  |
| **Site** |  | 0.975 |
| Face | 1.11 (2.15) |  |
| Scalp | 2.44 (4.64) |  |
| Neck | 0.4 (0.548) |  |
| **Hyperplasia** |  | 0.682 |
| No | 1.33 (3.02) |  |
| Yes | 1.8 (2.44) |  |

**Supplementary table 12.** The differences of QoL in different groups by gender, size, site

| **CDLQI questions** | **Gender** | | | | **Size** | | | **Site** | | |
| --- | --- | --- | --- | --- | --- | --- | --- | --- | --- | --- |
|  | **Male (n=17)** | **Female (n=26)** | **p** | **<1% (n=36)** | | **>=1% (n=7)** | **p** | **Head (n= 38)** | **Neck (n=5)** | **p** |
| **Q1. Itchy, 'scratchy', sore or painful skin** |  |  | 0.639 |  | |  | 0.671 |  |  | 0.475 |
| **Mean (SD)** | 0.18 (0.53) | 0.08 (0.27) |  | 0.11 (0.40) | | 0.14 (0.38) |  | 0.13 (0.41) | 0 (0) |  |
| **Q2. Embarrassed, self-conscious, upset or sad because of skin** |  |  | 0.941 |  | |  | 0.884 |  |  | 1 |
| **Mean (SD)** | 0.24 (0.56) | 0.23 (0.51) |  | 0.22 (0.49) | | 0.29 (0.76) |  | 0.24 (0.54) | 0.2 (0.45) |  |
| **Q3. Friendships** |  |  | 0.075 |  | |  | 0.317 |  |  | 0.315 |
| **Mean (SD)** | 0.35 (0.61) | 0.12 (0.43) |  | 0.17 (0.45) | | 0.43 (0.79) |  | 0.24 (0.54) | 0 (0) |  |
| **Q4. Clothing decisions** |  |  | 0.348 |  | |  | 0.098 |  |  | 0.414 |
| **Mean (SD)** | 0.24 (0.56) | 0.12 (0.43) |  | 0.08 (0.28) | | 0.57 (0.98) |  | 0.18 (0.51) | 0 (0) |  |
| **Q5. Going out, playing, or hobbies** |  |  | 0.179 |  | |  | 0.199 |  |  | 0.361 |
| **Mean (SD)** | 0.24 (0.44) | 0.12 (0.43) |  | 0.11 (0.32) | | 0.43 (0.79) |  | 0,18 (0.46) | 0 (0) |  |
| **Q6. Swimming or other sports** |  |  | 0.139 |  | |  | 0.062 |  |  | 0.474 |
| **Mean (SD)** | 0.18 (0.39) | 0.04 (0.20) |  | 0.06 (0.23) | | 0.29 (0.49) |  | 0.11 (0.31) | 0 (0) |  |
| **Q7. Schoolwork/Holiday in past week** |  |  | 0.844 |  | |  | 0.017 * |  |  | 0.548 |
| **Mean (SD)** | 0.06 (0.24) | 0.08 (0.27) |  | 0.03 (0.17) | | 0.29 (0.49) |  | 0.08 (0.27) | 0 (0) |  |
| **Q8. Calling names, teasing, bullying, asking questions, or avoiding** |  |  | 0.067 |  | |  | 0.005 ** |  |  | 0.414 |
| **Mean (SD)** | 0.26 (0.44) | 0.08 (0.39) |  | 0.06 (0.23) | | 0.57 (0.79) |  | 0.16 (0.44) | 0 (0) |  |
| **Q9. Sleep** |  |  | 0.083 |  | |  | 0.191 |  |  | 0.64 |
| **Mean (SD)** | 0.18 (0.53) | 0 (0) |  | 0.03 (0.17) | | 0.29 (076) |  | 0.08 (0.36) | 0 (0) |  |
| **Q10. Effect of treatment on quality of life** |  |  | 0.151 |  | |  | 0.479 |  |  | 0.955 |
| **Mean (SD)** | 0.29 (0.47) | 0.12 (0.33) |  | 0.17 (0.38) | | 0.29 (0.49) |  | 0.18 (0.39) | 0.2 (0.45) |  |

**Supplementary figure 1.** The geographical distribution of 130 SN patients


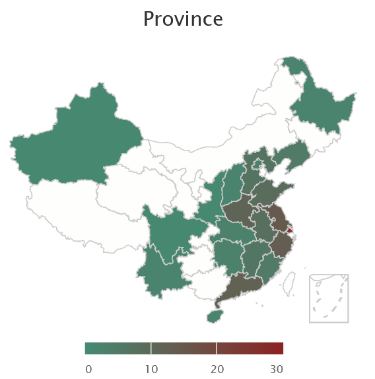


**Supplementary figure 2.** The family trees of 3 epidermal nevus history positive families


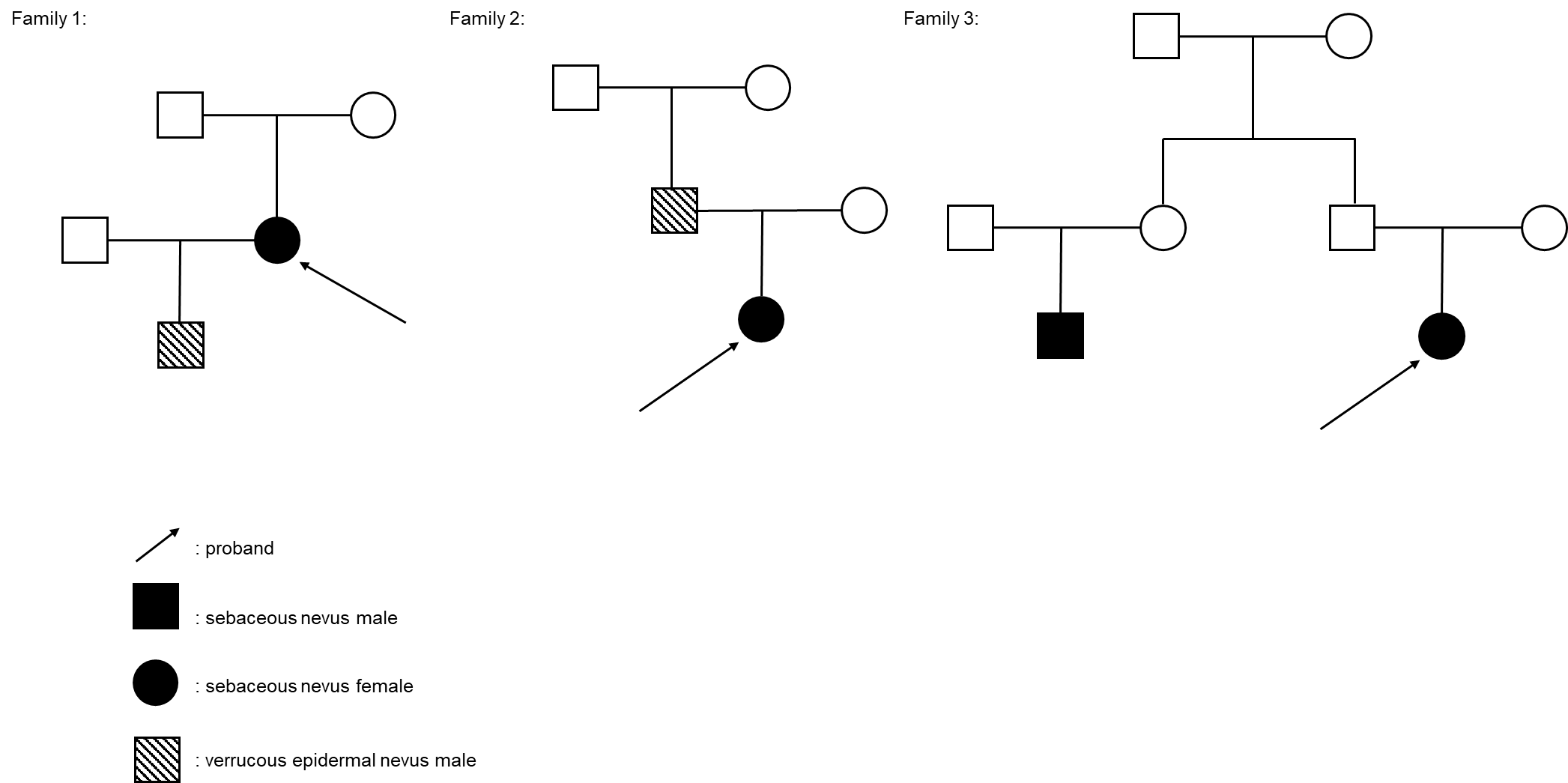


**Supplementary figure 3.** Photos of Family 1.


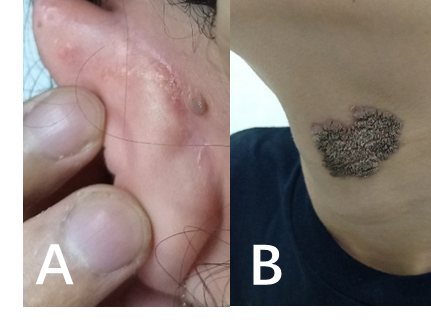


A. The linear sebaceous nevus behind ears of F1 mother.

B. The verrucous epidermal nevus on neck of F1 son

**Supplementary figure 4.** Photos of lesion of Case 2 and Case4

A. Case2 pre-treatment lateral view of the face: SN around eye and mouth.


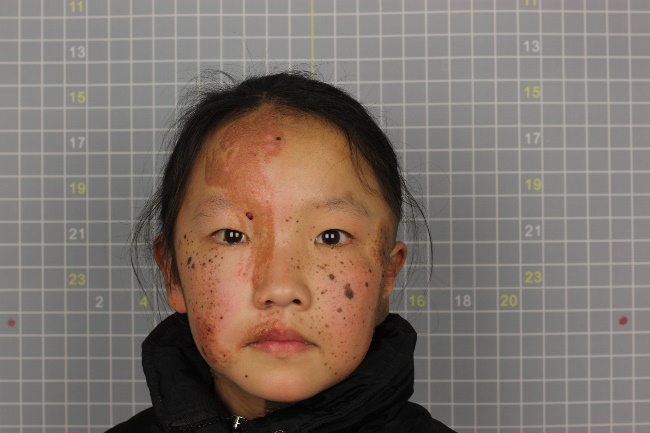

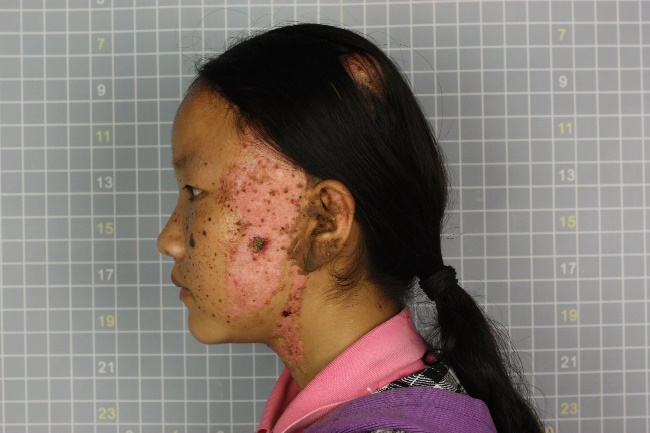

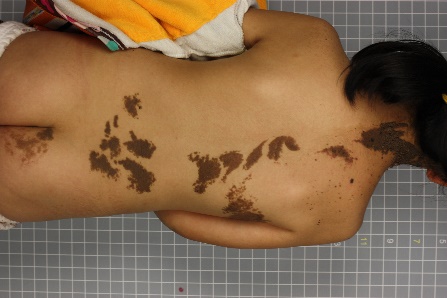

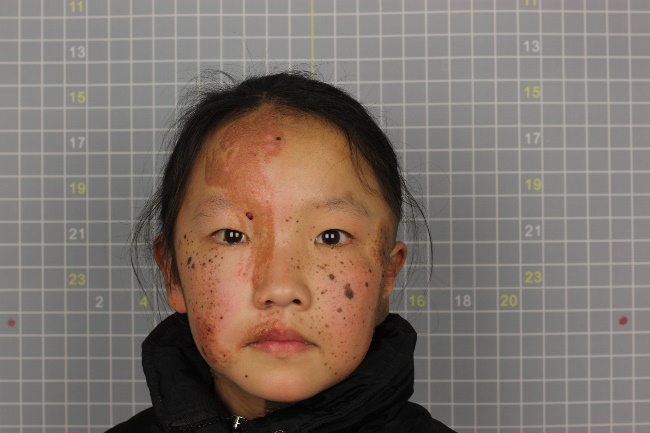

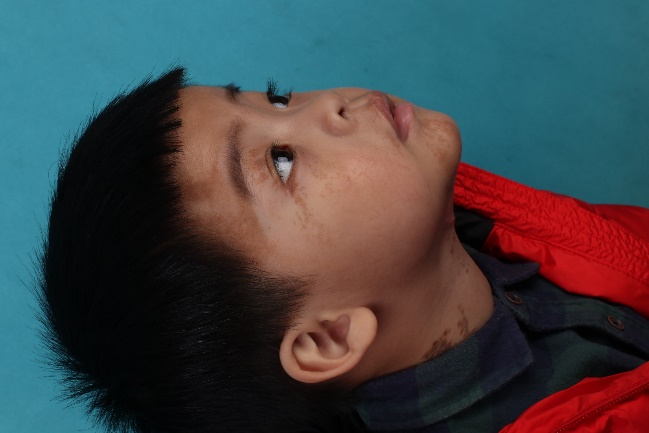


**C**


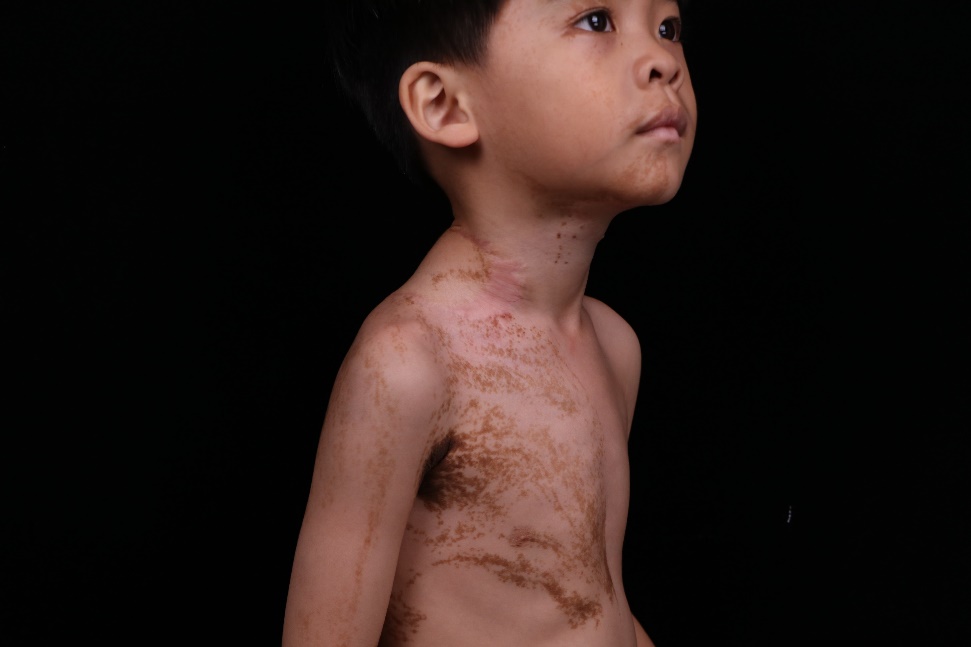


**B**

**D**

**E**

**F**

**A**

B. Case2 pre-treatment lateral view of the body: massive VEN.

C. Case4, post-first treatment anterior view: forehead SN.

D. Case4, post-first treatment lateral view: scalp SN.

E. Mild scoliosis of Case4 and body VEN.

F. The right eye of Case4 with abnormal ocular pigmentation

**Supplementary figure 5.** Histological characteristics of lesion from Case5.


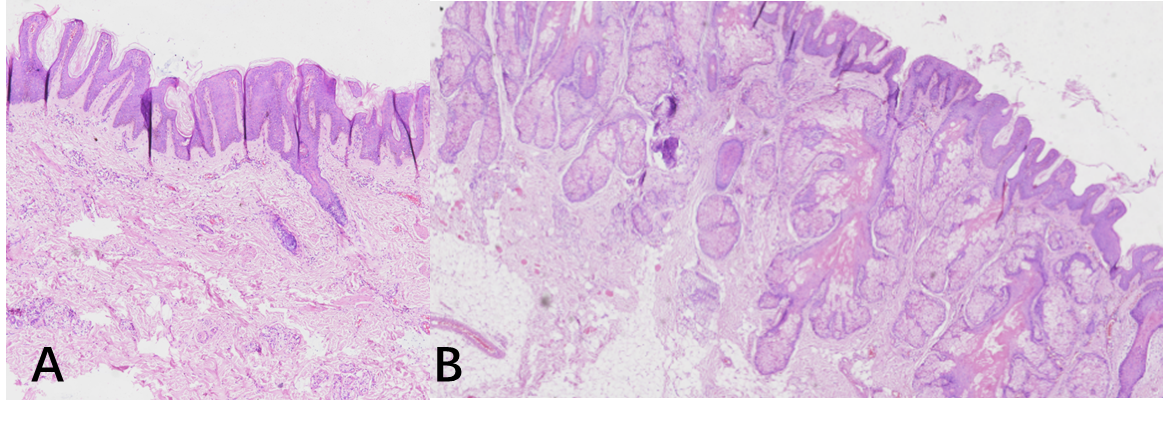


A. Histological characteristics of the peripheral part of lesion: epidermal hyperplasia and elongation of epidermal ridges.

B. Histological characteristics of the central part of lesion: sebaceous gland and epidermal hyperplasia and elongation of epidermal ridges
